# Cosmetic operations and Khovanov multicurves

**DOI:** 10.1007/s00208-023-02697-5

**Published:** 2023-09-11

**Authors:** Artem Kotelskiy, Tye Lidman, Allison H. Moore, Liam Watson, Claudius Zibrowius

**Affiliations:** 1 Department of Mathematics, Stony Brook University, Stony Brook, USA; 2 Department of Mathematics, North Carolina State University, Raleigh, USA; 3 Department of Mathematics and Applied Mathematics, Virginia Commonwealth University, Richmond, USA; 4 Department of Mathematics, University of British Columbia, Vancouver, Canada; 5 Department of Mathematical Sciences, Durham University, Durham, United Kingdom

## Abstract

We prove an equivariant version of the Cosmetic Surgery Conjecture for strongly invertible knots. Our proof combines a recent result of Hanselman with the Khovanov multicurve invariants ${\widetilde{{{\,\textrm{Kh}\,}}}}$ and ${\widetilde{{{\,\textrm{BN}\,}}}}$. We apply the same techniques to reprove a result of Wang about the Cosmetic Crossing Conjecture and split links. Along the way, we show that ${\widetilde{{{\,\textrm{Kh}\,}}}}$ and ${\widetilde{{{\,\textrm{BN}\,}}}}$ detect if a Conway tangle is split.

## Introduction

Here are two classical open conjectures in low dimensional topology:

### Cosmetic Surgery Conjecture

Given a non-trivial knot $K\subset S^3$ and $r,r'\in {\mathbb {Q}}\text {P}^1$, suppose there exists an orientation-preserving diffeomorphism $S^3_r(K)\cong S^3_{r'}(K)$. Then $r=r'$.

### Cosmetic Crossing Conjecture

**Fig. 1:**
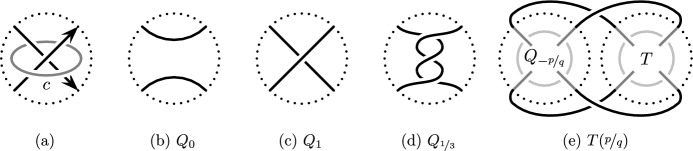
A crossing circle *c* (**a**), some examples of rational tangles (**b**–**d**), and the $\nicefrac {p}{q}$-rational filling $T(\nicefrac {p}{q})$ of a Conway tangle *T* (**e**)

Any crossing change that preserves the isotopy class of a knot must occur at a nugatory crossing, meaning the crossing circle (see Fig. 1a) bounds an embedded disk in the complement of the knot.

Two distinct rational Dehn surgeries along a knot are called *purely cosmetic* (or just cosmetic) if they yield diffeomorphic oriented three-manifolds. A single Dehn surgery along a knot may also be referred to as a ‘cosmetic surgery’ when it preserves the oriented diffeomorphism type of the three-manifold. Similarly, a crossing change that preserves the oriented isotopy class of a knot is called a *cosmetic crossing change*; likewise, a cosmetic tangle replacement is one that preserves the oriented isotopy type of the knot. We expect that the Cosmetic Crossing Conjecture holds even for unoriented isotopy types. In fact, the results in this paper are true for unoriented knots. All knots in this paper are unoriented by default.

The Cosmetic Surgery Conjecture originates with Gordon [8, Conjecture 6.1] and the Cosmetic Crossing Conjecture is due to Lin. Both problems appear in Kirby’s Problem List [14, Problem 1.81 A, Bleiler; Problem 1.58, Lin]. This paper explores what Khovanov homology can say about these conjectures from the perspective of the multicurve technology developed by Kotelskiy, Watson, and Zibrowius [15].

### Cosmetic surgeries

Following Sakuma [29], a strongly invertible knot is a pair (*K*, *h*), where *K* is a knot in $S^3$ and *h* is an orientation-preserving involution of $S^3$ mapping *K* to itself and reversing a choice of orientation of *K*. Strongly invertible knots (*K*, *h*) and $(K',h')$ are equivalent if there exists an orientation-preserving diffeomorphism *f* on $S^3$ for which $f(K)=K'$ (so that *K* and $K'$ are equivalent knots) and $h = f^{-1}\circ h'\circ f$. Any strong inversion *h* on a knot $K\subset S^3$ restricts to the elliptic involution of the torus on the boundary of the knot exterior, and hence can be extended to an involution $h_r$ on $S^3_r(K)$ for any slope $r\in {\mathbb {Q}}\text {P}^1$. (The latter statement can be found in [10]. See also [1] for a nice exposition.) This extension is unique up to homotopy.

In this article we prove the following equivariant version of the Cosmetic Surgery Conjecture:

#### Theorem 1.1

Given a non-trivial strongly invertible knot (*K*, *h*) and $r,r'\in {\mathbb {Q}}\text {P}^1$, suppose that there exists an orientation-preserving diffeomorphism $f: S^3_r(K)\rightarrow S^3_{r'}(K)$ such that $h_{r'}\circ f= f \circ h_r$. Then $r=r'$.

A strongly invertible knot (*K*, *h*) gives rise to a Conway (ie four-ended) tangle $T \subset B^3$ constructed as follows. Waldhausen showed that the fixed point set ${\text {Fix}}(h)$ in $S^3$ is an unknot intersecting *K* in two points [36], and thus restricts to a pair of arcs in the knot exterior $X_K=S^3\smallsetminus \nu (K)$. (This is a special case of the well-known Smith Conjecture, which is now a theorem.) Therefore, taking the quotient produces a Conway tangle $T={\text {Im}}({\text {Fix}}(h)\cap X_K)$ inside the three-ball $B^3=X_K / h$. For an illustration, see [39, Figure 4].

Cosmetic surgeries along a strongly invertible knot (*K*, *h*) are closely related to cosmetic tangle fillings of the quotient tangle *T*, due to the Montesinos trick [22]: The fixed point set of the involution $h_{\nicefrac {p}{q}}$ on $S^3_{\nicefrac {p}{q}}(K)$ restricted to the surgery solid torus is a pair of arcs, which descends in the quotient of the solid torus to a trivial Conway tangle. Montesinos gives an explicit correspondence between the rational surgery slope and a rational parameterization of the trivial tangle in the quotient. In particular, $S^3_{\nicefrac {p}{q}}(K)$ is the two-fold branched cover of the rational tangle filling $T(\nicefrac {p}{q}) = Q_{\nicefrac {-p}{q}}\cup T$ illustrated in Fig. 1, where $Q_{\nicefrac {-p}{q}}$ denotes the rational tangle of slope $\nicefrac {-p}{q}$ and the tangle *T* is the quotient tangle of (*K*, *h*). Conversely, a cosmetic crossing change on a knot induces a cosmetic surgery on the two-fold branched cover. Hence, the two cosmetic conjectures are related, and the analogue of Theorem 1.1 is:

#### Theorem 1.2

Let *T* be a Conway tangle in the three-ball with an unknot closure. Suppose *T*(*r*) and $T(r')$ are isotopic links in the three-sphere for some $r, r' \in {\mathbb {Q}}\text {P}^1$. Then $r = r'$ or *T* is rational.

Indeed Theorem 1.2 is equivalent to Theorem 1.1 by taking two-fold branched covers. The condition of having an unknot closure is equivalent to considering surgeries on knots in $S^3$ and non-triviality of the knot in $S^3$ is equivalent to the tangle not being rational.

Gordon and Luecke’s solution to the knot complement problem implies that no non-trivial knots admit cosmetic surgeries when one of the slopes is $\infty$ [7]. Boyer and Lines showed that if $\Delta ''_K(1)\ne 0$, rational surgeries along *K* are always distinct, where $\Delta _K(t)$ is the Alexander polynomial [3, Proposition 5.1], and a similar result may be formulated in terms of the Jones polynomial [12]. Note that the obstructions of [3, 12] do not disqualify strongly invertible knots from admitting cosmetic surgeries because all Alexander polynomials of knots can be realized by strongly invertible knots [28].

Strong restrictions on cosmetic surgeries can be formulated in terms of Heegaard Floer homology, as shown in work of Wang [37], Ozsváth and Szabó [25], and Ni and Wu [23]. Using immersed curves, Hanselman’s work [9] extends these results; in particular, he shows that cosmetic surgery slopes have to be either $\pm 2$ or $\nicefrac {\pm 1}{n}$. In an entirely different direction, hyperbolic geometry techniques have been used to obtain other restrictions on the set of cosmetic filling slopes (namely in terms of their lengths) [6]. Using the strategies above and others, the Cosmetic Surgery Conjecture has been established for knots of genus one, cables, connected sums and three-braids [32, 33, 35, 37].

Up to now, Khovanov homology has remained conspicuously absent from this story. Our proof of Theorem 1.2 relies on the Khovanov multicurve technology developed by Kotelskiy, Watson, and Zibrowius [15, 17]. This theory assigns an immersed multicurve in the four-punctured sphere to a Conway tangle and a suitable Lagrangian Floer homology of these multicurves computes the Khovanov homology.

### Cosmetic crossings

As illustrated by the proof of Theorem 1.2 in Sect. 4, the immersed curve invariants for Khovanov homology provide a powerful tool for studying the behavior of Khovanov homology under tangle fillings. Note that the Cosmetic Crossing Conjecture can be viewed in terms of comparing the $+1$ and $-1$ tangle fillings of a Conway tangle; hence, the Khovanov tangle invariants provide a natural tool for studying the Cosmetic Crossing Conjecture. In fact, Theorem 1.2 immediately implies the Cosmetic Crossing Conjecture for the unknot, originally due to Scharlemann–Thompson [31]. In general, the Cosmetic Crossing Conjecture is still open, but has been established for knots which are two-bridge [34] or fibered [13, 27], as well as for large classes of knots which are genus one [2, 11] or alternating [18]. In the second half of this paper, we illustrate the utility of this theory by giving elementary proofs of two other known results about the Cosmetic Crossing Conjecture.

First, we recall that there is a generalization of the Cosmetic Crossing Conjecture. Let *c* be a crossing circle for a knot *K* as in Fig. 1a. Then, performing $\nicefrac {-1}{n}$-Dehn surgery on *c* produces a new knot $K_n$ which corresponds to adding *n* full right-handed twists at the crossing. The Generalized Cosmetic Crossing Conjecture predicts that if *c* is a non-nugatory crossing, then $K_n$ is not isotopic to $K_m$ for $n \ne m$.

We first give a new proof of a recent result of Wang on the Generalized Cosmetic Crossing Conjecture, which also used Khovanov homology:

#### Theorem 1.3

(Wang [38]) Let *K* be a knot obtained by a non-trivial band surgery on a split link *L*. If $K_n$ is obtained by inserting $n \in {\mathbb {Z}}$ twists into the band, then $K_n$ is not isotopic to $K_m$ for any $n \ne m$.

We also prove that the generalized crossing conjecture holds “asymptotically”:

#### Theorem 1.4

Let *K* be an unoriented knot and *c* a crossing circle for a non-nugatory crossing. Let $\{K_n\}_{n \in {\mathbb {Z}}}$ be the associated sequence of knots obtained by inserting twists at *c*. Then there exists an integer *N* such that $\{K_n\}_{|n|\ge N}$ are pairwise different.

#### Remark 1.5

While we could not find Theorem 1.4 written explicitly in the literature, it is certainly known to experts using standard techniques from three-manifold topology. Rather than give this alternative proof here in full, we illustrate this by sketching a proof for a suitably generic case. Suppose that $K \cup c$ is a hyperbolic link. Then $K_n$ is obtained by performing $\nicefrac {-1}{n}$-surgery on *c*. Hence, $K_n$ is hyperbolic for all but finitely many *n* and further the hyperbolic volume of $K_n$ converges to the hyperbolic volume of $K \cup c$, which is strictly greater than that of any $K_n$. The asymptotics of this convergence is described by the work of Neumann–Zagier [24] and precludes having more than finitely many knots in the sequence of fixed volume. It is interesting that Khovanov homology and the hyperbolic volume establish the same result in this setting. We note that the behaviour of the Jones polynomial under twisting in relation with hyperbolic geometry has been considered, see [5] for example.

Along the way, we establish the following technical result about the Khovanov multicurve invariants, which may be of independent interest:

#### Theorem 1.6

The invariants ${\widetilde{{{\,\textrm{Kh}\,}}}}(T)$ and ${\widetilde{{{\,\textrm{BN}\,}}}}(T)$ detect if the Conway tangle *T* is split.

Note that Theorem 1.6 has been established for the analogous knot Floer homology multicurve invariant by Lidman, Moore, and Zibrowius [19].

### Outline

In Sect. 2, we give the requisite background on the immersed curve invariants for tangles. In Sect. 3, we prove Theorem 1.6. In Sect. 4, we prove Theorem 1.2 (and hence Theorem 1.1). In Sect. 5, we prove Theorems 1.3 and 1.4.

## Review of the Khovanov multicurve invariants

In this section, we review some properties of the immersed curve invariants ${\widetilde{{{\,\textrm{Kh}\,}}}}$ and ${\widetilde{{{\,\textrm{BN}\,}}}}$ of pointed Conway tangles from [15]. We work exclusively over the field ${\mathbb {F}}$ of two elements and only summarize those properties that we will need in this paper; more elaborate introductions highlighting different aspects of the invariants can be found in [16, 17].

Let *T* be an oriented *pointed* Conway tangle, that is a four-ended tangle in the three-ball $B^3$ with a choice of distinguished tangle end, which we mark by $*$. Denote by $S^2_{4,*}$ the four-punctured sphere $\partial B^3\smallsetminus \partial T$; the puncture marked by $*$ will be called *special*. We associate with such a tangle *T* invariants ${\widetilde{{{\,\textrm{BN}\,}}}}(T)$ and ${\widetilde{{{\,\textrm{Kh}\,}}}}(T)$ that take the form of multicurves on $S^2_{4,*}$. By multicurve, we mean a collection of immersed curves that carry certain extra data. Broadly speaking, there are two kinds of such curves: compact and non-compact. A compact immersed curve in $S^2_{4,*}$ is an immersion of $S^1$, considered up to regular homotopy, that (up to conjugation) defines a primitive element of $\pi _1(S^2_{4,*})$, and each of these curves is decorated with a local system, ie an invertible matrix over ${\mathbb {F}}$ considered up to matrix similarity. A non-compact immersed curve in $S^2_{4,*}$ is a non-null-homotopic immersion of an interval, with ends on the three non-special punctures of $S^2_{4,*}$; see [15, Definition 1.4]. Non-compact curves do not carry local systems. In addition, all curves are equipped with a bigrading; more on this in Sect. 2.3 below.

### Remark 2.1

**Fig. 2:**
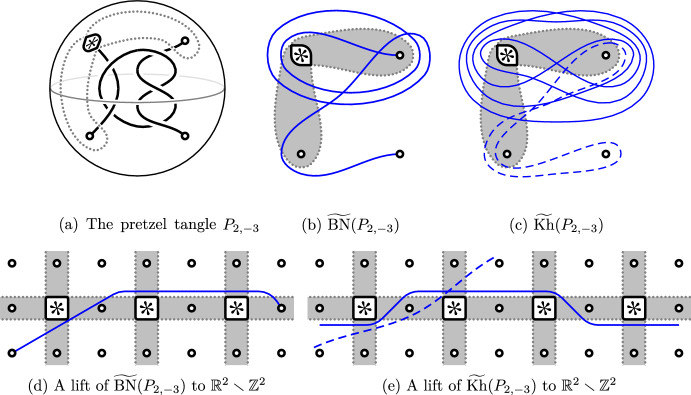
The multicurve invariants for the pretzel tangle $P_{2,-3}$. Under the covering ${\mathbb {R}}^2\smallsetminus {\mathbb {Z}}^2\rightarrow S^2_{4,*}$, the shaded regions in (**b** + **c**) correspond to the shaded regions in (**d** + **e**)

We often draw $S^2_{4,*}$ as the plane plus a point at infinity minus the four punctures. To help identify this abstract surface with $\partial B^3\smallsetminus \partial T$, we then add two dotted gray arcs that parametrize the surface, see Fig. 2a–c. The blue curves in these figures show the multicurves ${\widetilde{{{\,\textrm{BN}\,}}}}(P_{2,-3})$ and ${\widetilde{{{\,\textrm{Kh}\,}}}}(P_{2,-3})$ for the pretzel tangle $P_{2,-3}$; cf [15, Example 6.7]. All components of these curves carry the (unique) one-dimensional local system.

### The construction of the multicurves

The starting point is the algebraic tangle invariant $[\![ T ]\!]_{/l}$ due to Bar-Natan. The invariant $[\![ T ]\!]_{/l}$ is a chain complex over a certain cobordism category, whose objects are crossingless tangle diagrams [4]; we refer to [15, Section 2] for a detailed introduction to complexes over categories/algebras, and the equivalent viewpoint through type D structures. In [15, Theorem 1.1], it was shown that any such complex can be rewritten as a chain complex  over the following category ${{\,\mathrm{{\mathcal {B}}}\,}}$, consisting of two objects and morphisms equal to paths in a quiver modulo relations:
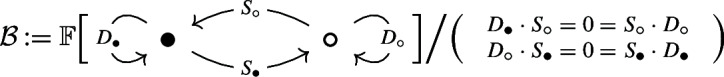


Here, the objects  and  correspond to the crossingless tangles  and , respectively.

We will refer to ${{\,\mathrm{{\mathcal {B}}}\,}}$ as a (quiver) algebra, and to  as a chain complex over the algebra ${{\,\mathrm{{\mathcal {B}}}\,}}$. Defining $D{:}{=} D_{\bullet } + D_{\circ }$ and $S{:}{=} S_{\bullet } + S_{\circ }$ often allows us to drop the subscripts of the algebra elements of ${{\,\mathrm{{\mathcal {B}}}\,}}$. The chain homotopy type of  is an invariant of the tangle *T*. Moreover, using the central element

$$\begin{aligned} H{:}{=} D+S^2 = D_{\bullet } + D_{\circ } + S_{\circ }S_{\bullet } + S_{\bullet }S_{\circ } ~ \in ~ {{\,\mathrm{{\mathcal {B}}}\,}}\end{aligned}$$

we define a chain complex  as the mapping conewhere $H\cdot {{\,\textrm{id}\,}}$ is the endomorphism of  defined by $x\xrightarrow {H}x$ for all generators *x* of . The chain homotopy type of  is also a tangle invariant.

**Fig. 3:**
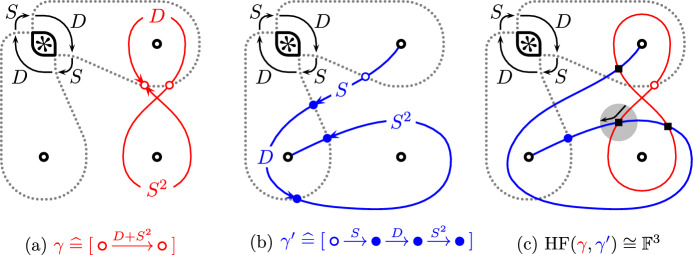
Two immersed curves and their corresponding chain complexes (**a** + **b**) and their Lagrangian Floer homology (**c**); cf [15, Examples 1.6 and 1.7]

The multicurve invariants ${\widetilde{{{\,\textrm{BN}\,}}}}(T)$ and ${\widetilde{{{\,\textrm{Kh}\,}}}}(T)$ are geometric interpretations of  and  respectively, made possible by the following classification result: The homotopy equivalence classes of chain complexes over ${{\,\mathrm{{\mathcal {B}}}\,}}$ are in one-to-one correspondence with multicurves on the four-punctured sphere $S^2_{4,*}$ [15, Theorem 1.5]. In a little more detail, this correspondence (which we denote by $\mathrel {\widehat{=}}$) uses the parametrization of $S^2_{4,*}$ given by the two dotted arcs described in Remark 2.1. We will generally assume that the multicurves intersect these arcs minimally. Then, roughly speaking, the intersection points correspond to generators of the associated chain complexes and paths between those intersection points correspond to the differentials. The two examples in Fig. 3 should give the reader a general impression how this works.

#### Example 2.2

For the trivial tangle , the chain complex  consists of a single object  and the differential vanishes. The corresponding multicurve  consists of a single vertical arc connecting the two non-special tangle ends. The chain complex  and the corresponding curve ${\widetilde{{{\,\textrm{Kh}\,}}}}(Q_\infty )$ is shown in Fig. 3a. The local system on this curve is one-dimensional.

The tangle  is obtained from the trivial tangle $Q_\infty$ by adding three twists to the two lower tangle ends. Its invariant ${\widetilde{{{\,\textrm{BN}\,}}}}(Q_{\nicefrac {1}{3}})$ is shown in Fig. 3b. Note that it agrees with the vertical arc ${\widetilde{{{\,\textrm{BN}\,}}}}(Q_{\infty })$ up to three twists. This is not a coincidence; one can show that adding twists to *any* tangle (not just a rational tangle) corresponds to adding twists to the multicurves; see [15, Theorem 1.13]. Thus, the identification of $\partial B^3\smallsetminus \partial T$ with the abstract surface $S^2_{4,*}$ containing the multicurves is natural.

**Fig. 4:**
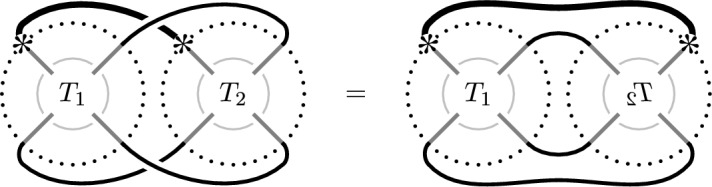
Two tangle decompositions defining the link $T_1\cup T_2$. The tangle  is the result of rotating $T_2$ around the vertical axis. By rotating the entire link on the right-hand side around the vertical axis, we can see that $T_1\cup T_2=T_2\cup T_1$

### A gluing theorem

The multicurve invariants satisfy various gluing formulas [15, Theorem 1.9]. The one that we will use in this paper is the following:

#### Theorem 2.3

Let $L=T_1\cup T_2$ be the result of gluing two oriented pointed Conway tangles as in Fig. 4 such that the orientations match. Let ${\text {m}}$ be the map identifying the two four-punctured spheres. Then

$$\begin{aligned} {\widetilde{{{\,\textrm{Kh}\,}}}}(L) \cong {\text {HF}}\left( {\text {m}}({\widetilde{{{\,\textrm{Kh}\,}}}}(T_1)),{\widetilde{{{\,\textrm{BN}\,}}}}(T_2)\right) \cong {\text {HF}}\left( {\text {m}}({\widetilde{{{\,\textrm{BN}\,}}}}(T_1)),{\widetilde{{{\,\textrm{Kh}\,}}}}(T_2)\right) \end{aligned}$$

The Lagrangian Floer homology  between two curves  and  is a vector space that can be computed as follows. First, we draw the curves in such a way that minimizes the number of intersection points between  and .  is then equal to a vector space freely generated by those intersection points, provided that the curves are not homotopic to each other [15, Theorem 5.25]. (We will always be able to make this assumption in this paper.) For instance, with Example 2.2 and Fig. 3 in mind, the Khovanov homology of the trefoil can be computed as follows:Finally, the Lagrangian Floer homology between two multicurves is simply the direct sum of the Lagrangian Floer homologies between individual components.

### Gradings

Khovanov homology is a *bigraded* homology theory, and this bigrading is often what makes it a powerful invariant. The multicurves ${\widetilde{{{\,\textrm{BN}\,}}}}(T)$ and ${\widetilde{{{\,\textrm{Kh}\,}}}}(T)$ also carry bigradings. We now describe how the gradings work: first on the algebra ${{\,\mathrm{{\mathcal {B}}}\,}}$, then on chain complexes, then on multicurves, and then finally on Lagrangian Floer homology between multicurves.

Equip the algebra ${{\,\mathrm{{\mathcal {B}}}\,}}$ with quantum grading *q*, which is determined by

$$\begin{aligned} q(D_{\bullet }) = q(D_{\circ }) = -2 \quad \text {and} \quad q(S_{\bullet }) = q(S_{\circ }) = -1. \end{aligned}$$

The homological grading is defined to be 0 for all elements of ${{\,\mathrm{{\mathcal {B}}}\,}}$.

Differentials of bigraded chain complexes over ${{\,\mathrm{{\mathcal {B}}}\,}}$ are required to preserve quantum grading and increase the homological grading by 1. Concretely this means that if a differential contains a morphism $x \xrightarrow {a}y$ (where *x* and *y* are generators of the complex and $a\in {{\,\mathrm{{\mathcal {B}}}\,}}$), then $q(a)+q(y)-q(x)=0$ and $h(a)+h(y)-h(x)=1$. We often specify the quantum gradings  of generators of such complexes via superscripts, like so: .

The bigrading on multicurves is simply a bigrading on the associated chain complex. To be more precise, let ${\mathcal {G}}(\Gamma )$ be the set of intersection points between a multicurve $\Gamma$ and the two parametrizing arcs. A bigrading on the multicurve $\Gamma$ is a map

$$\begin{aligned} \psi : {\mathcal {G}}(\Gamma ) \rightarrow {\mathbb {Z}}^2 \end{aligned}$$

satisfying certain compatibility conditions; namely, if *X* is the bigraded vector space freely generated by the elements *x* in ${\mathcal {G}}(\Gamma )$ over ${\mathbb {F}}$ with bigrading , then the differential on *X* corresponding to $\Gamma$ is required to preserve the quantum grading and to increase the homological grading by 1. These conditions can be stated in terms of the geometry of the multicurves as in [17, 19]. However, in this paper, we will only need the above algebraic formulation.

If *T* is an unoriented tangle, the bigradings on ${\widetilde{{{\,\textrm{BN}\,}}}}(T)$ and ${\widetilde{{{\,\textrm{Kh}\,}}}}(T)$ are only well-defined as relative bigradings, that is to say, the map $\psi$ is only well-defined up to adding a constant map. To fix this overall shift, an orientation of *T* is required; see for example [4, Definition 6.4] or [15, Proposition 4.8].

Let  and  be two bigraded multicurves and suppose  and  are the corresponding bigraded chain complexes over ${{\,\mathrm{{\mathcal {B}}}\,}}$. Then  also carries a bigrading. It can be computed using the fact that this vector space is bigraded isomorphic to the homology of the morphism space  [15, Theorem 1.5] (see the discussion before [15, Definition 2.4] for the definition of the differential on a morphism space between two complexes). The quantum grading of a morphism , where $\alpha \in {{\,\mathrm{{\mathcal {B}}}\,}}$, is computed using the formula ; an analogous formula holds for the homological grading. Each intersection point generating  corresponds to a morphism from which we can read off the bigrading [16, Section 7]. For instance, the highlighted intersection point in Fig. 3c corresponds to the morphism , so the bigrading of this intersection point is equal toThe ${\mathbb {Z}}/2$ reduction of the homological grading corresponds to the usual grading on Lagrangian Floer homology; for example, the gradings of two intersection points connected by a bigon differ by 1. The quantum grading plays a similar role to the Alexander grading in Heegaard Floer homology in that it corresponds to counting the multiplicities of connecting domains near tangle ends, similar to [19, Sections 3.5 and 3.6]. For the grading computations in this paper, however, we only use the above formulation of the bigrading in terms of morphism spaces.

### Geography of components of ${\widetilde{{{\,\textrm{Kh}\,}}}}$

We now recall some basic facts about ${\widetilde{{{\,\textrm{Kh}\,}}}}(T)$ and ${\widetilde{{{\,\textrm{BN}\,}}}}(T)$ from [15, Section 6]. In this paper, we will focus only on tangles without closed components. For such tangles, ${\widetilde{{{\,\textrm{BN}\,}}}}(T)$ consists of a single non-compact component and a (possibly zero) number of compact components. In contrast, ${\widetilde{{{\,\textrm{Kh}\,}}}}(T)$ consists of compact components only.

Often, multicurves become easier to manage when considered in a certain covering space of $S^2_{4,*}$, namely the planar cover that factors through the toroidal two-fold cover:

$$\begin{aligned} ({\mathbb {R}}^2 \smallsetminus {\mathbb {Z}}^2) \rightarrow (T^2 \smallsetminus 4{\text {pt}}) \rightarrow S^2_{4,*}\end{aligned}$$

This is illustrated in Fig. 2 for the multicurve invariants of the pretzel tangle $P_{2,-3}$.

**Fig. 5:**
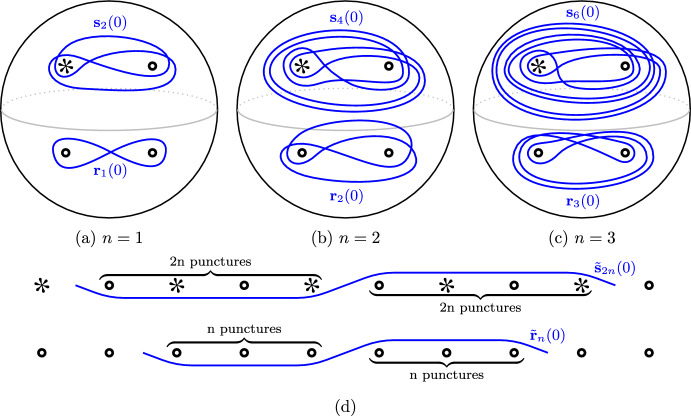
The curves ${\textbf{r}}_n(0)$ and ${\textbf{s}}_{2n}(0)$ (**a**–**c**) and their lifts to ${\mathbb {R}}^2\smallsetminus {\mathbb {Z}}^2$ (**d**). While not visually apparent, the curves ${\textbf{r}}_n(0)$ are invariant under the Dehn twist interchanging the lower two punctures

#### Definition 2.4

Given an immersed curve $c \looparrowright S^2_{4,*}$, denote by ${\tilde{c}}$ a lift of $c$ to the cover ${\mathbb {R}}^2\smallsetminus {\mathbb {Z}}^2$. For $n\in {{\mathbb {N}}}$, let ${\textbf{r}}_{n}(0)$ and ${\textbf{s}}_{2n}(0)$ be the immersed curves in $S^2_{4,*}$ that respectively admit lifts to the curves ${\tilde{{\textbf{r}}}}_{n}(0)$ and ${\tilde{{\textbf{s}}}}_{2n}(0)$ in Fig. 5d; curves for $n=1,2,3$ are illustrated in Fig. 5a–c. For every $\nicefrac {p}{q}\in {\mathbb {Q}}\text {P}^1$, we respectively define the curves ${\textbf{r}}_n(\nicefrac {p}{q})$ and ${\textbf{s}}_{2n}(\nicefrac {p}{q})$ as the images of ${\textbf{r}}_{n}(0)$ and ${\textbf{s}}_{2n}(0)$ under the action of

$$\begin{aligned} \begin{bmatrix} q &{}\quad r \\ p &{}\quad s \end{bmatrix} \end{aligned}$$

considered as an element of the mapping class group fixing the special puncture ${{\,\textrm{Mod}\,}}(S^2_{4,*}) \cong {{\,\textrm{PSL}\,}}(2,{\mathbb {Z}})$, where $qs-pr=1$. (This transformation maps straight lines of slope 0 to straight lines of slope $\nicefrac {p}{q}$.) We call ${\textbf{r}}_{n}(\nicefrac {p}{q})$ a curve of *rational type, slope* $\nicefrac {p}{q}$, *and length* *n*. We call ${\textbf{s}}_{2n}(\nicefrac {p}{q})$ a curve of *special type, slope* $\nicefrac {p}{q}$, *and length* 2*n*. The local systems on all these curves are defined to be trivial.

The following classification result is [17, Theorem 6.5].

#### Theorem 2.5

For any pointed Conway tangle *T*, every component of ${\widetilde{{{\,\textrm{Kh}\,}}}}(T)$ is equal to ${\textbf{r}}_n(\nicefrac {p}{q})$ or ${\textbf{s}}_{2n}(\nicefrac {p}{q})$ for some $n\in {{\mathbb {N}}}$ and $\nicefrac {p}{q}\in {\mathbb {Q}}\text {P}^1$, up to some bigrading shift. In other words, components of ${\widetilde{{{\,\textrm{Kh}\,}}}}(T)$ are completely classified by their type, slope, length, and bigrading.

As already mentioned in Example 2.2, the multicurve invariants are natural with respect to adding twists; these twists generate ${{\,\textrm{Mod}\,}}(S^2_{4,*})$. Thus, the invariant ${\widetilde{{{\,\textrm{Kh}\,}}}}(Q_{\nicefrac {p}{q}})$ of a $\nicefrac {p}{q}$-rational tangle $Q_{\nicefrac {p}{q}}$ is equal to ${\textbf{r}}_1(\nicefrac {p}{q})$, justifying the terminology. In fact, we have the following detection result [17, Theorem 5.7].

#### Theorem 2.6

A pointed Conway tangle *T* is rational if and only if ${\widetilde{{{\,\textrm{Kh}\,}}}}(T)$ consists of a single component ${\textbf{r}}_1(\nicefrac {p}{q})$ for some $\nicefrac {p}{q}\in {\mathbb {Q}}\text {P}^1$.

Rational components can also occur in the invariants of non-rational tangles. In fact, if *T* has no closed component, we know that there is always at least one such component [17, Corollary 6.42]. For example, the curve ${\widetilde{{{\,\textrm{Kh}\,}}}}(P_{2,-3})$ from Fig. 2c consists of the special component ${\textbf{s}}_4(0)$ and the rational component ${\textbf{r}}_1(\nicefrac {1}{2})$. Such rational components detect how tangle ends are connected [17, Theorem 6.41]:

#### Theorem 2.7

Suppose a pointed Conway tangle *T* has connectivity . Then the slope $\nicefrac {p}{q}\in {\mathbb {Q}}\text {P}^1$ (with *p* and *q* coprime) of any odd-length rational component of ${\widetilde{{{\,\textrm{Kh}\,}}}}(T)$ satisfies $p\equiv 0\mod 2$.

#### Remark 2.8

Components of the invariant ${\widetilde{{{\,\textrm{BN}\,}}}}(T)$, even compact ones, can be much more complicated than components of ${\widetilde{{{\,\textrm{Kh}\,}}}}(T)$; for an example, see [17, Figure 26]. However, the invariants of rational tangles are very simple: Any lift of ${\widetilde{{{\,\textrm{BN}\,}}}}(Q_{\nicefrac {p}{q}})$ to ${\mathbb {R}}^2\smallsetminus {\mathbb {Z}}^2$ is a straight line segment of slope $\nicefrac {p}{q}$ connecting the lifts of two non-special punctures.

### A dimension formula

#### Definition 2.9

Given two slopes $\nicefrac {p}{q},\nicefrac {p'}{q'}\in {\mathbb {Q}}\text {P}^1$, where (*p*, *q*) and $(p',q')$ are pairs of mutually prime integers, define the distance between two slopes as

#### Lemma 2.10

Let $s,r\in {\mathbb {Q}}\text {P}^1$ be two distinct slopes. Let ${\textbf{a}}_{s}{:}{=}{\widetilde{{{\,\textrm{BN}\,}}}}(Q_{s})$ and let $\gamma$ be a rational or special curve of length $\ell$ and slope *r*. Then

$$\begin{aligned} \dim {\text {HF}}({\textbf{a}}_{s},\gamma ) = \ell \cdot \Delta (s,r) \end{aligned}$$

Our proof of Lemma 2.10 makes use of the following basic idea, on which many other calculations in this paper rely as well.

#### Observation 2.11

The number of intersection points between two curves $\gamma _1$ and $\gamma _2$ in $S^2_{4,*}$ is equal to the number of intersection points between the preimage $\Gamma _1$ of $\gamma _1$ and any lift ${\tilde{\gamma }}_2$ of $\gamma _2$ in ${\mathbb {R}}^2 \smallsetminus {\mathbb {Z}}^2$, provided the endpoints of ${\tilde{\gamma }}_2$ are disjoint from $\Gamma _1$.

#### Proof of Lemma 2.10

Write $s=\nicefrac {p}{q}$ and $r=\nicefrac {p'}{q'}$ for pairs (*p*, *q*) and $(p',q')$ of mutually prime integers. Observe that $\dim {\text {HF}}({\textbf{a}}_{s},\gamma )$ stays invariant under changing the parametrization of the four-punctured sphere. So let us apply the linear transformation corresponding to the matrixwhere $n,m\in {\mathbb {Z}}$ are such that $mp'-nq'=1$. This transformation maps $\gamma$ to a curve of slope 0 and ${\textbf{a}}_s$ to a curve of slope $\tfrac{p'q-q'p}{nq-mp}$. The distance between these curves remains the same. This shows that if the formula holds for the case $r=0$, then it also holds in general. So suppose $r=0$. Then $\Delta (s,r)=|p|$, which by assumption is non-zero. We need to see that in this case

$$\begin{aligned} \dim {\text {HF}}({\textbf{a}}_s,\gamma ) = \ell \cdot |p| \end{aligned}$$

**Fig. 6:**
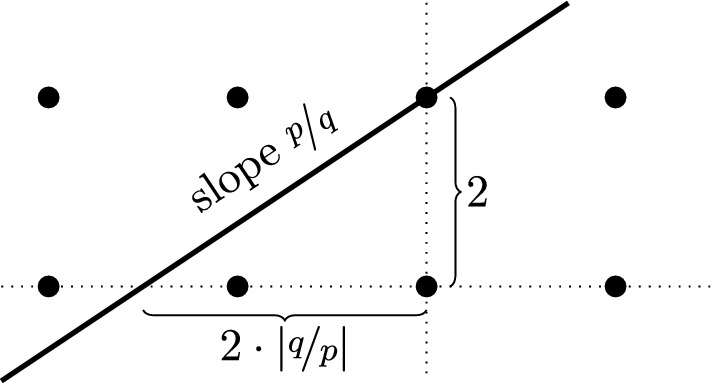
The set ${\textbf{A}}_s$ in the proof of Lemma 2.10 is the union of the bold straight line with all its $2{\mathbb {Z}}\times 2{\mathbb {Z}}$-translates

This identity can be easily checked in the covering space ${\mathbb {R}}^2\smallsetminus {\mathbb {Z}}^2$ using Observation 2.11. If ${\tilde{\gamma }}$ is a lift of $\gamma$, $\dim {\text {HF}}({\textbf{a}}_s,\gamma )$ is equal to the number of times that ${\tilde{\gamma }}$ intersects the preimage ${\textbf{A}}_s$ of ${\textbf{a}}_s$ (assuming the endpoints of ${\tilde{\gamma }}$ are disjoint from ${\textbf{A}}_s$). This is the same as the number of intersection points of ${\textbf{A}}_s$ with certain horizontal straight line segments of length $2\ell$ (whose endpoints are disjoint from ${\textbf{A}}_s$). So it suffices to show that the distance between consecutive intersection points of ${\textbf{A}}_s$ with any straight horizontal line is constant equal to $\nicefrac {2}{|p|}$. A simple geometric argument, illustrated in Fig. 6, shows that said distance is the minimum of all non-zero expressions of the formwhere *a* and *b* vary over all integers. The claim now follows from Bézout’s identity and the assumption that *p* and *q* are coprime. $\square$

## Splitness detection for ${\widetilde{{{\,\textrm{BN}\,}}}}$ and ${\widetilde{{{\,\textrm{Kh}\,}}}}$

**Fig. 7:**
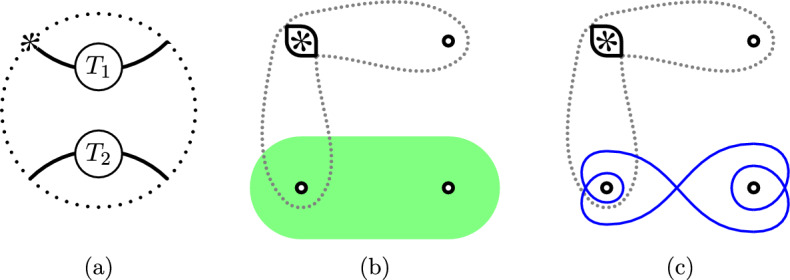
**(a)** A split tangle and **(b)** the region in $S^2_{4,*}$ supporting the multicurve invariants of any such tangle, according to Theorem 3.1. **(c)** Illustrates the generalized figure-eight curves ${\textbf{e}}_n(0)$ for $n=2$; note that ${\textbf{e}}_1(0)={\textbf{r}}_1(0)$

Recall that a Conway tangle $T\subset B^3$ is *split* if there exists an essential curve in $\partial B^3 \smallsetminus \partial T$ that bounds a disk in $B^3 \smallsetminus T$. If the slope of this curve is 0, we call the tangle *horizontally split*. Equivalently, a tangle is horizontally split if it can be written as a disjoint union of two-ended tangles $T_1$ and $T_2$ as in Fig. 7a. In this section, we show that ${\widetilde{{{\,\textrm{Kh}\,}}}}$ and ${\widetilde{{{\,\textrm{BN}\,}}}}$ detect this property:

### Theorem 3.1

For any Conway tangle *T* the following conditions are equivalent:

- *T* is horizontally split;
- Up to some bigrading shift, each component of ${\widetilde{{{\,\textrm{BN}\,}}}}(T)$ is equal to the horizontal arc  or a generalized figure-eight curve 
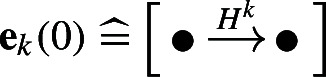
 for some $$k>0$$;
- Up to some bigrading shift, each component of ${\widetilde{{{\,\textrm{Kh}\,}}}}(T)$ is equal to ${\textbf{r}}_1(0)$;
- Up to homotopy, ${\widetilde{{{\,\textrm{BN}\,}}}}(T)$ is entirely contained in the shaded region in Fig. 7b;
- Up to homotopy, ${\widetilde{{{\,\textrm{Kh}\,}}}}(T)$ is entirely contained in the shaded region in Fig. 7b;
- In the homotopy equivalence class  there is a representative chain complex that contains no generator ;
- In the homotopy equivalence class  there is a representative chain complex that contains no generator .

### Remark 3.2

There is an analogous detection result for the Heegaard Floer tangle invariant ${{\,\textrm{HFT}\,}}$ [19, Theorem 4.1].

By the naturality of ${\widetilde{{{\,\textrm{BN}\,}}}}$ and ${\widetilde{{{\,\textrm{Kh}\,}}}}$ under twisting [15, Theorem 1.13], it follows that a tangle is split if and only if ${\widetilde{{{\,\textrm{Kh}\,}}}}(T)$ consists of rational components of the same slope, or equivalently, if and only if ${\widetilde{{{\,\textrm{BN}\,}}}}(T)$ consists only of generalized figure-eight curves and arcs of the same slope.

### Proof

We start with the implication (1) $\Rightarrow$ (2a). Bar-Natan associates with the two-ended tangles $T_1$ and $T_2$ the invariants $[\![ T_1 ]\!]_{/l}$ and $[\![ T_2 ]\!]_{/l}$, which are chain complexes over the cobordism category whose objects are crossingless two-ended tangles. Thanks to delooping [15, Observation 4.18], we can write these as complexes over the subcategory generated by the trivial tangle . The morphisms in this subcategory can be represented by linear combinations of cobordisms without closed components, ie identity cobordisms with some number of handles attached. By Bar-Natan’s gluing formalism, $[\![ T ]\!]_{/l}$ is then a tensor product of the complexes $[\![ T_1 ]\!]_{/l}$ and $[\![ T_2 ]\!]_{/l}$. In particular, the objects of $[\![ T ]\!]_{/l}$ are equal to  and the differential consists of linear combinations of identity cobordisms with some number of handles attached to one of the components. Since we are working with coefficients in ${\mathbb {F}}$, the (4*Tu*)-relation [15, Definition 4.3] allows us to move all handles on these cobordisms to the component containing the basepoint $*$ of . Attaching a handle to the component with a basepoint corresponds to multiplying by *H* [15, Definition 4.10], so $[\![ T ]\!]_{/l}$ is a chain complex over the graded algebra ${\mathbb {F}}[H]$. Therefore, up to homotopy,  is a direct sum of complexes of the form 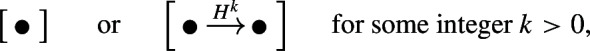


as required. (This follows from essentially the same arguments as the classification of free chain complexes over a principal ideal domain; see for instance [26, Propositions A.4.3 and A.8.1].)

The implication (2a)$\Rightarrow$(2b) follows from the definition of ${\widetilde{{{\,\textrm{Kh}\,}}}}(T)$ as the curve corresponding to the mapping cone  of the identity map on  multiplied by *H*. The equivalences (3a)$\Leftrightarrow$ (4a) and (3b)$\Leftrightarrow$ (4b) and the implications (2a) $\Rightarrow$ (3a) and (2b) $\Rightarrow$ (3b) are obvious. The equivalence (4a)$\Leftrightarrow$ (4b) follows from the observation that any complex  corresponding to a curve ${\widetilde{{{\,\textrm{BN}\,}}}}(T)$ contains a generator  if and only if the same is true for its mapping cone .

**Fig. 8:**
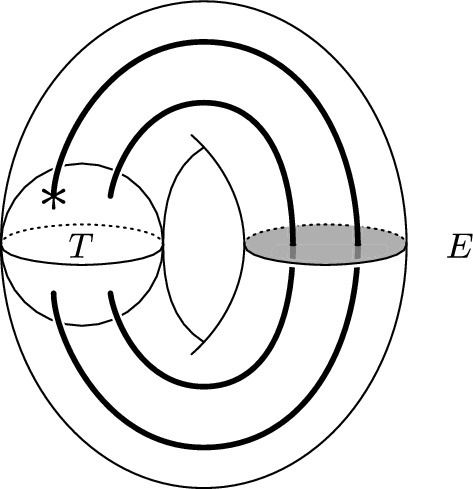
The annular link $T_a(\infty )$ in $S^1\times D^2$. The shaded disk on the right shows the essential disk *E* used in the proof of Lemma 3.3

The implication (4a)$\Rightarrow$(1) remains. This direction relies on a detection result for annular Khovanov homology; this was established by Xie using annular instanton homology. We know that there is a complex  representing ${\widetilde{{{\,\textrm{BN}\,}}}}(T)$ that only contains generators . This is equivalent to saying that the tangle invariant $[\![ T ]\!]_{/l}$, as a homotopy equivalence class of chain complexes over , has a representative containing only generators . Let $T_a(\infty )$ be the annular link shown in Fig. 8. Its annular Khovanov homology ${{\,\textrm{AKh}\,}}(T_a(\infty );{\mathbb {F}})$ can be computed from $[\![ T ]\!]_{/l}$ via gluing arguments similar to [4, Section 5]. It is concentrated in annular grading zero, because in that computation, every circle has winding number zero around the annulus. By the classification of finitely generated Abelian groups, we can write

$$\begin{aligned} {{\,\textrm{AKh}\,}}(T_a(\infty );{\mathbb {Z}}) \cong {\mathbb {Z}}^n \oplus \Big ( \bigoplus _{i=1}^m {\mathbb {Z}}/2^{\ell _i}{\mathbb {Z}}\Big ) \oplus T_{\text {odd}} \end{aligned}$$

for some integers $n,\ell _1,\ldots ,\ell _m$, where $T_{\text {odd}}$ is the subgroup of elements of ${{\,\textrm{AKh}\,}}(T_a(\infty );{\mathbb {Z}})$ of odd order. Hence, by the universal coefficient theorem,

$$\begin{aligned} {{\,\textrm{AKh}\,}}(T_a(\infty );{\mathbb {C}}) \cong {{\,\textrm{AKh}\,}}(T_a(\infty );{\mathbb {Z}})\otimes {\mathbb {C}}\oplus \underbrace{{\text {Tor}}^{\mathbb {Z}}({{\,\textrm{AKh}\,}}(T_a(\infty );{\mathbb {Z}}),{\mathbb {C}})}_{\cong 0} \cong {\mathbb {C}}^n \end{aligned}$$

and

$$\begin{aligned} {{\,\textrm{AKh}\,}}(T_a(\infty );{\mathbb {F}}) \cong {{\,\textrm{AKh}\,}}(T_a(\infty );{\mathbb {Z}})\otimes {\mathbb {F}}\oplus {\text {Tor}}^{\mathbb {Z}}({{\,\textrm{AKh}\,}}(T_a(\infty );{\mathbb {Z}}),{\mathbb {F}}) \cong ({\mathbb {F}}^n\oplus {\mathbb {F}}^m) \oplus {\mathbb {F}}^m. \end{aligned}$$

Therefore, if ${{\,\textrm{AKh}\,}}(T_a(\infty );{\mathbb {F}})$ is concentrated in annular grading zero, then so is ${{\,\textrm{AKh}\,}}(T_a(\infty );{\mathbb {C}})$. We can now apply Xie’s detection result [40, Corollary 1.6] to deduce that the link $T_a(\infty )$ is contained in a three-ball embedded in the solid torus $S^1\times D^2$. We conclude with Lemma 3.3 below. $\square$

### Lemma 3.3

If $T_a(\infty )$ is contained in a three-ball inside a solid torus, then *T* must be horizontally split.

### Proof

Without loss of generality, we may assume that *T* has no unlinked closed components.

Let *S* be the boundary of the three-ball containing $T_a(\infty )$ and let *E* be the essential disk in $S^1\times D^2$ shown in Fig. 8. Without loss of generality, we may assume that *S* intersects *E* transversely, so that $E\cap S$ is a union of circles. Clearly, $E\cap S\ne \varnothing$ since *S* separates $E\cap T_a(\infty ) \ne \varnothing$ from $\partial E$. We now consider those circles as subsets of *S*. One of them, let us call it *C*, is innermost, so it bounds a disk $D_S$ in *S* disjoint from any other circles. The circle *C* also bounds a disk $D_E$ in *E*.

Suppose $D_E$ is disjoint from $T_a(\infty )$. Then $D_E\cup D_S$ is disjoint from $T_a(\infty )$ and bounds a three-ball *B* in the solid torus. Since *T* has no unlinked closed component, *B* is disjoint from $T_a(\infty )$. Therefore, there exists an isotopy of *S* that is the identity outside a small neighbourhood of *B* and inside this neighbourhood removes the circle *C* from $E\cap S\ne \varnothing$ as well as any other component of $E\cap S\ne \varnothing$ that lies in $D_E$.

After repeating this procedure a finite number of times, we may assume that $D_E$ intersects $T_a(\infty )$ non-trivially. Since the sphere $D_S\cup D_E$ intersects $T_a(\infty )$ in an even number of points and $D_S$ is disjoint from $T_a(\infty )$, $D_E$ must contain both intersection points of *E* with $T_a(\infty )$. So $E\smallsetminus D_E$ is an annulus and $D_S\cup (E\smallsetminus D_E)$ is an essential disk which, after isotopy, certifies that *T* is a horizontally split tangle. $\square$

### Remark 3.4

Lemma 3.3 and its proof generalize to tangles with arbitrarily many tangle ends as follows: A tangle is split if its $\infty$-closure in the solid torus is contained in a three-ball. However, it need not be horizontally split. For an example, consider the tangle .

## Proof of Theorem 1.2

The two-fold branched cover $\Sigma (T)$ of $B^3$ branched over *T* is the exterior of a knot $K\subset S^3$. Suppose, without loss of generality, that $T(\infty )$ is the unknot. Then, a curve of slope $\infty$ on the boundary of the three-ball containing *T* lifts to a meridian of *K*. By adding the appropriate number of twists on the right of the tangle *T*, we can further assume that *T* is parametrized such that a curve of slope 0 lifts to a longitude of *K*.

Suppose now that $T(r)\cong T(r')$ as unoriented links and that the tangle *T* is non-rational. It suffices to show that $r=r'$. Assume, for sake of contradiction, that $r\ne r'$. The identity $T(r)\cong T(r')$ implies

$$\begin{aligned} S^3_r(K)= \Sigma (T(r))\cong \Sigma (T(r'))=S^3_{r'}(K). \end{aligned}$$

We now apply Hanselman’s theorem [9, Theorem 2] to deduce that $r=-r'$ where

$$\begin{aligned} r=\pm 2 \text { (case 1)} \quad \text {or} \quad r=\nicefrac {\pm 1}{n} \text { for some positive integer }n \text { (case 2)}. \end{aligned}$$

The hypothesis of Hanselman’s theorem is indeed satisfied: If *K* were the unknot, then there would be a unique strong inversion, and its quotient *T* would be a rational tangle, contradicting our assumptions.

As a consequence of our chosen parametrization, the connectivity of the tangle *T* is . This can be seen as follows: First, observe that *T* has no closed component and the connectivity of the tangle is not . Both follow from the fact that $T(\infty )$ is the unknot. Secondly, if *T*(0) were a knot, $\det T(0)=|H_1(\Sigma (T(0)))|$ would be odd, so its two-fold branched cover $\Sigma (T(0))$ would be a rational homology sphere. This contradicts the fact that the two-fold branched cover is 0-surgery on the knot $K\subset S^3$. So *T*(0) is a two-component link and hence the connectivity of *T* is not .

The strategy for the remainder of the proof is to show that the reduced Khovanov homologies ${\widetilde{{{\,\textrm{Kh}\,}}}}(T(r))$ and ${\widetilde{{{\,\textrm{Kh}\,}}}}(T(r'))$ are distinct. We first equip *T*(*r*) and $T(r')$ with orientations such that they agree as oriented links. Then ${\widetilde{{{\,\textrm{Kh}\,}}}}(T(r))$ and ${\widetilde{{{\,\textrm{Kh}\,}}}}(T(r'))$ agree as absolutely bigraded groups. We work with coefficients in ${\mathbb {F}}$, so that reduced Khovanov homology is independent of the reduction point [30, Corollary 3.2.C]. We will compute ${\widetilde{{{\,\textrm{Kh}\,}}}}(T(r))$ and ${\widetilde{{{\,\textrm{Kh}\,}}}}(T(r'))$ by pairing the ${\widetilde{{{\,\textrm{BN}\,}}}}$-invariants of the rational tangle fillings (arcs) with the multicurve . A priori, the absolute bigrading on  depends on the orientation of the tangle, but as we will see below, it is in fact orientation independent. Since $T(\infty )$ is the unknot, we know that  has only one intersection with the vertical arc . Special curves ${\textbf{s}}_{2n}(s)$ intersect ${\textbf{a}}_\infty$ in more than one point, unless they have slope $s=\infty$, in which case they are disjoint from ${\textbf{a}}_\infty$. Similarly, a rational curve ${\textbf{r}}_{n}(s)$ intersects ${\textbf{a}}_\infty$ in more than one point, unless $n=1$ and $s\in {{\mathbb {Z}}}$, in which case there is a single intersection point. Hence, we may writewhere  are special components of slope $\infty$ and , of slope $s\in {{\mathbb {Z}}}$. Since *T* is non-rational, $m>0$ by Theorem 2.6. Moreover, by Theorem 2.7, we know that *s* is an even integer.

We now consider the two cases separately. The arguments in both cases are essentially the same. We first show that the slope *s* of  must be 0 for the total dimensions of ${\widetilde{{{\,\textrm{Kh}\,}}}}(T(r))$ and ${\widetilde{{{\,\textrm{Kh}\,}}}}(T(r'))$ to agree; then we compute the absolute quantum gradings and observe that they are different.

**Case 1:** $\{r,r'\}=\{2,-2\}$. Since the connectivity of the tangle *T* is , $T(+2)=T(-2)$ is a link with two components. Consider their linking number. If we choose the same orientation of the tangle *T*, the linking numbers of $T(+2)$ and $T(-2)$ are different, since the crossings in the $\pm 2$-twist tangles then have different signs. So up to an overall orientation reversal (which does not affect the reduced Khovanov homology), we may assume that the orientations on $T(+2)$ and $T(-2)$ are as follows:
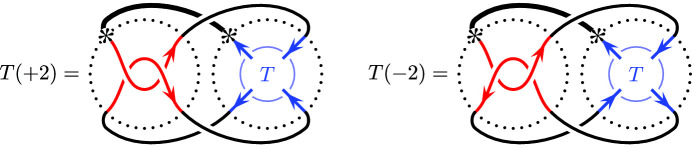
 Since the crossings in the $\pm 2$-twist tangles are all positive, the linking number of the tangle *T* with the orientation as in $T(+2)$ is the same as with the orientation as in $T(-2)$. (The linking number of a tangle is defined in [15, Definition 4.7].) Since the two orientations are obtained by reversing one strand, these linking numbers also differ by a sign, so the linking number of *T* is zero. Hence  is independent of orientations; see for example [15, Proposition 4.8].

Define two arcs


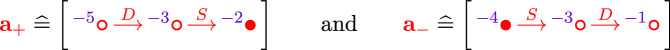

**Fig. 9:**
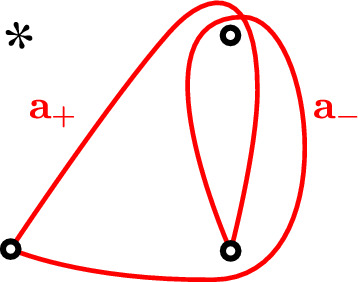
The arcs  and

These are the arc invariants of the mirrors of the $\pm 2$-twist tangles in $T(+2)$ and $T(-2)$, respectively, and are illustrated in Fig. 9. (For instance, this calculation follows from [15, Example 4.27], using (1) the relation $q=2(h+\delta )$ between the $\delta$-, homological, and quantum gradings in Khovanov theory, and (2) the formula from [15, Proposition 4.8] for the grading shift induced by reversing the orientation of a tangle component.) Then, by the pairing theorem,The total dimensions of the first *m* pairs of summands are identical:This is because all intersection points lie within a small neighbourhood of a vertical line through the special puncture and  and  look identical in this neighbourhood. By Lemma 2.10, and because a figure eight and an arc of the same slope intersect minimally in two points, the dimensions of the final summands  areOur assumption that $T(+2)\cong T(-2)$ implies that the dimensions of reduced Khovanov homology agree, so the slope $s=0$.

**Fig. 10:**
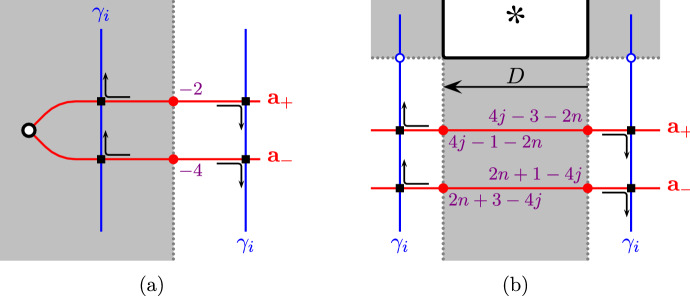
Canonical generators of  in the proof of Theorem 1.2, **(a)** Case 1 and **(b)** Case 2. The arrows indicate the corresponding morphisms used for computing the gradings, as in Sect. 2.3

We now consider the quantum gradings. Recall that the grading on  is independent of the orientation on . Since $T(\infty )$ is the unknot, the quantum gradings on  and  agree. Thus,  and  are graded isomorphic, because both are graded isomorphic to the reduced Khovanov homology of the same oriented Hopf link. Moreover, for all $i=1,\ldots ,m$, the quantum grading is shifted such thatwhich together with the previous observation contradicts ${\widetilde{{{\,\textrm{Kh}\,}}}}(T(+2))\cong {\widetilde{{{\,\textrm{Kh}\,}}}}(T(-2))$. The grading shift for  can be seen as follows: After pulling the curves  sufficiently tight, their intersections with the arcs  are all in a small neighbourhood of the intersection points of  with the parametrizing arcs corresponding to the generators  as shown in Fig. 10a from the viewpoint of the planar cover of $S^2_{4,*}$. The arcs  are parallel in this region, so the grading difference is precisely the (negative of) the grading difference between these two generators.

**Case 2:** $\{r,r'\}=\{\nicefrac {1}{n}, -\nicefrac {1}{n}\}$. Since the connectivity of the tangle *T* is , $T(\nicefrac {1}{n})=T(\nicefrac {-1}{n})$ is a knot and up to overall orientation reversal, the orientations on $T(\nicefrac {1}{n})$ and $T(\nicefrac {-1}{n})$ are as follows:DefineNote that $j=1,\ldots ,\lfloor \tfrac{n}{2}\rfloor$. The arcs  and  are the ${\widetilde{{{\,\textrm{BN}\,}}}}$ invariants of the mirrors of the $\nicefrac {\pm 1}{n}$-twist tangles in $T(\nicefrac {1}{n})$ and $T(\nicefrac {-1}{n})$, respectively; see [15, Example 6.2, Proposition 4.8]. So as in Case 1, the pairing theorem allows us to writeThe total dimensions of the first *m* pairs of summands are identical, regardless of the slope *s* of the rational component . Since the slope *s* is an even integer, it never agrees with $\nicefrac {\pm 1}{n}$, so the dimensions of the final summands  are equal to $|1\mp sn|$ by Lemma 2.10 and hence only agree if $s=0$.

We now compute quantum gradings. First,  agree as absolutely bigraded homology groups, since they compute the reduced Khovanov homology of an unknot, shifted in quantum grading by the same amount. To compute the grading shifts for the first *m* summands, we observe that after pulling the curves  sufficiently tight (see [17, Definition 6.1] and the discussion afterwards), the intersection points between the arcs  and  sit in a small neighbourhood of the vertical line through the special marked point. If *n* is even, the relevant portions of the complexes  and  are, respectively,


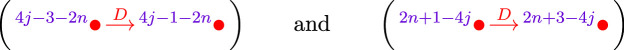


where $j=1,\ldots ,\lfloor \tfrac{n}{2}\rfloor$. The corresponding curve segments are illustrated in Fig. 10b. They are obviously parallel, so there is a one-to-one correspondence between generators  and generators  such that the quantum gradings satisfy

$$\begin{aligned} q(x_-)-q(x_+)=8j-4-4n<0 \end{aligned}$$

for $j=1,\ldots ,\lfloor \tfrac{n}{2}\rfloor$. If *n* is odd, there are additional generators stemming from the generators  and  of the complexes . The corresponding curve segments look as in Fig. 10a, except that the quantum gradings are different. The correspondence from the case that *n* is even extends to the case that *n* is odd so that the quantum gradings of the additional generators satisfy

$$\begin{aligned} q(x_-)-q(x_+)=-2<0 \end{aligned}$$

The grading shifts are strictly negative in all cases, contradicting ${\widetilde{{{\,\textrm{Kh}\,}}}}(T(\nicefrac {1}{n}))\cong {\widetilde{{{\,\textrm{Kh}\,}}}}(T(\nicefrac {-1}{n}))$. $\square$

### Remark 4.1

This proof highlights the utility of the quantum gradings in Khovanov homology. However, it also suggests an alternate strategy that avoids gradings in these complexes altogether through the exact triangle. In general, by using the immersed curves reformulation of Khovanov homology, one is able to split up the skein exact triangle into several summands. There is one for each component of the immersed multicurve for the tangle complementary to the crossing where the exact triangle is being implemented. For each exact triangle, the dimensions of the three groups are simply computed by a count of intersections between the component and three rational curves in the four-punctured sphere of distance one. This gives much stronger constraints on the structure of the exact triangle. Frequently, as a result, the maps in the exact triangle can be computed as well, and additional grading structures can be deduced. A similar perspective on these exact sequences is seen in bordered Heegaard Floer homology for three-manifolds with torus boundary [20, Section 11.2].

## The generalized Cosmetic Crossing Conjecture holds asymptotically

**Fig. 11:**
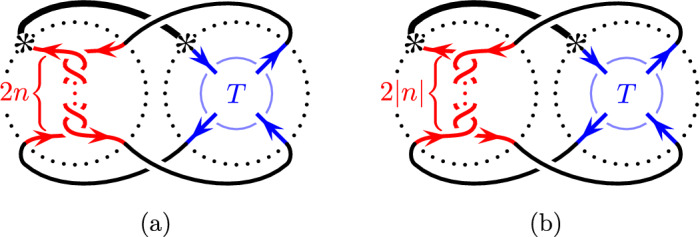
The knots $K_n$ for $n\ge 0$ (**a**) and $n\le 0$ (**b**)

Throughout this section we fix a Conway tangle *T* with connectivity  and without any closed components. Furthermore, we consider the family of knots $\{K_n\}_{n\in {\mathbb {Z}}}$ shown in Fig. 11 and defined by $K_n=T(\nicefrac {1}{2n})$ for $n\in {\mathbb {Z}}$. Equivalently, each knot $K_n$ is the result of a band surgery on a fixed two-component link *T*(0) and the knots $K_n$ and $K_{n+1}$ are obtained from each other by adding a full twist to the band. This point of view explains the restrictions placed on the tangle *T*.

If *T* is horizontally split, then all $K_n$ are equal to each other. We now restate the two conjectures on cosmetic crossings in terms of Conway tangles:

### Conjecture 5.1

(*Cosmetic Crossing Conjecture*) Suppose *T* is not horizontally split. Then $K_0$ and $K_1$ are different as unoriented knots.

### Conjecture 5.2

(*Generalized Cosmetic Crossing Conjecture*) Suppose *T* is not horizontally split. Then the unoriented knots $\{K_n\}_{n\in {\mathbb {Z}}}$ are pairwise different.

Recently, Wang showed that these conjectures hold assuming *T*(0) is a split link [38]. While he states these conjectures for oriented knots, we are not aware of any counterexamples to the conjectures as stated above for unoriented knots. Note, however, that a crossing change may result in the mirror of the original knot, as illustrated by the $(3,-3,\pm 1)$-pretzel knots, see the remarks to [14, Problem 1.58].

We now show that the generalized Cosmetic Crossing Conjecture holds “asymptotically”:

### Theorem 5.3

(Reformulation of Theorem 1.4) Suppose *T* is not horizontally split. Then there exists an integer *N* such that the knots $\{K_n\}_{|n|\ge N}$ are pairwise different as unoriented knots.

### Lemma 5.4

Suppose ${\widetilde{{{\,\textrm{Kh}\,}}}}(T)$ only contains curves of slope 0 of which at least one is special. Then $K_n\not \cong K_m$ for any $n\ne m$.

**Fig. 12:**
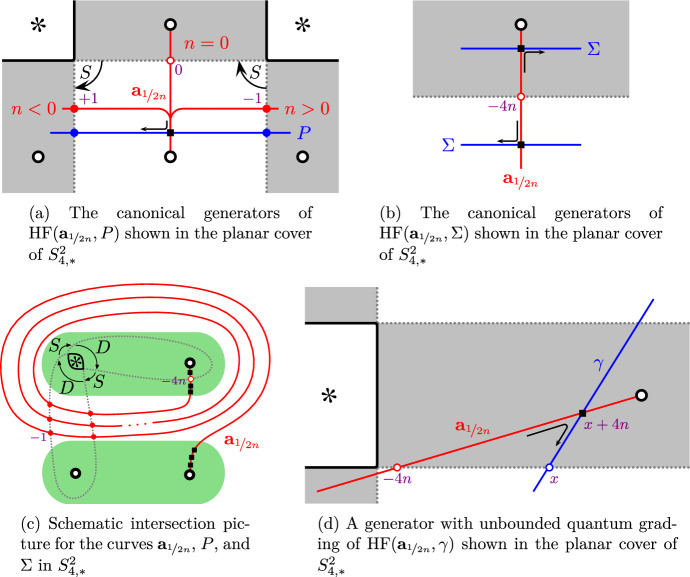
Illustration of intersection points between various curves in the proofs of Lemmas 5.4 and 5.5. Numbers near generators (intersection points) indicate their quantum gradings. In **(c)**, the dots $\bullet$ indicate the intersection points of the curve  with  (contained in the bottom region) and  (contained in the top region)

### Proof

Let us write , where  consists of special components and  of rational components. By assumption . Also , since the pairing of the arc  with ${\widetilde{{{\,\textrm{Kh}\,}}}}(T)$ computes the Khovanov homology of the link *T*(0) and as such is non-zero. Since the connectivity of the tangle *T* is , the induced orientation of $K_n$ on the tangle *T* and its rational filling are as shown in Fig. 11a, up to overall orientation reversal. Define the arc of slope $\nicefrac {1}{2n}$:


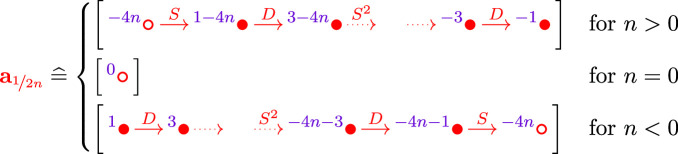


By the same calculation as in the proof of Theorem 1.2, Case 2 (using [15, Example 6.2, Proposition 4.8]), these are the arc invariants of the mirrors of the rational fillings of *T*. Then, by the pairing theorem,We now study how both summands behave when varying *n*; Fig. 12c depicts the schematic picture of intersections in $S^2_{4,*}$. After pulling the multicurves  and  sufficiently tight, the intersection points generating  all sit close to the end of  corresponding to the generator  for $n>0$,  for $n=0$, and  for $n<0$. The corresponding curve segments are shown in Fig. 12a. We see that the quantum grading of  is independent of *n*; see Sect. 2.3 for how gradings are computed.

We now investigate the pairing of  with special curves. After pulling all multicurves sufficiently tight, the intersection points generating  all sit close to the end of  corresponding to the generator , see Fig. 12b. Thus, the shift in quantum grading is as follows:Therefore $K_n\not \cong K_m$ if $n\ne m$. $\square$

### Lemma 5.5

Suppose ${\widetilde{{{\,\textrm{Kh}\,}}}}(T)$ contains a curve of a non-zero slope. Then there exists *N* such that the unoriented knots $\{K_n\}_{|n|\ge N}$ are all different.

### Proof

Let us write  for some integer $m>0$. For each $i=1,\dots ,m$, let $(p_i,q_i)$ be a pair of mutually prime integers such that the slope of  is $\nicefrac {p_i}{q_i}$. By assumption, there is some $i\in \{1,\ldots ,m\}$ such that $p_i\ne 0$. Let

$$\begin{aligned} M=\max \left\{ \left. \tfrac{|q_i|}{2|p_i|}\right| i\in \{1,\ldots ,m\}: p_i\ne 0\right\} \end{aligned}$$

Then for $|n|> M$, the slope $\nicefrac {1}{2n}$ of the curve  is distinct from $\nicefrac {p_i}{q_i}$. Therefore, by Lemma 2.10,where $\ell _i$ is the length of . As we have seen in the proof of Lemma 5.4, if $p_i=0$, the dimension of  is independent of *n*. If $p_i\ne 0$, the sign of the expression $\nicefrac {q_i}{p_i}-2n$ is the same for all $n>M$. Therefore, $\dim {\widetilde{{{\,\textrm{Kh}\,}}}}(K_n)$ is a strictly increasing function in *n* for $n>M$. The same argument shows that it is strictly decreasing for $n<-M$. Thus the knots $\{K_n\}_{n>M}$ are pairwise different, and so are the knots $\{K_n\}_{n<-M}$.

It remains to distinguish the two families. For this we first prove that the quantum grading of $\{{\widetilde{{{\,\textrm{Kh}\,}}}}(K_n)\}_{n\gg 0}$ is unbounded above and bounded below.

The existence of a lower bound follows from the following two observations: first, the quantum gradings of the generators of  are bounded above by $-1$. Second, every intersection point generating the Lagrangian Floer homology between  and a rational or special curve can be represented by a homogeneous morphism containing a component labelled by an algebra element of quantum grading greater than or equal to $-2$ (namely one of the algebra elements ${{\,\textrm{id}\,}},S,S^2, D\in {{\,\mathrm{{\mathcal {B}}}\,}}$); this follows from an elementary argument about straight lines in the covering space $S^2_{4,*}$. Thus, if the minimal grading of a generator of  is $\mu$, the formula from Sect. 2.3 for computing the quantum grading of generators of  gives us $\mu -(-1)+(-2)$ as a lower bound.

Next, we show that the quantum grading of $\{{\widetilde{{{\,\textrm{Kh}\,}}}}(K_n)\}_{n\gg 0}$ has no upper bound. By assumption, there exists a component  of  of non-zero slope. For $n\gg 0$, we may assume that the slope of  is bigger than the slope $\nicefrac {1}{2n}$ of the arc . Then, there exists an intersection point close to the generator , which looks like Fig. 12d. Clearly, the quantum gradings of this family of generators are unbounded.

Analogous arguments imply that the quantum grading of $\{{\widetilde{{{\,\textrm{Kh}\,}}}}(K_n)\}_{n\ll 0}$ is unbounded below and bounded above. This proves that there exists $N\gg M$ such that knots in $\{K_n\}_{n>N} \cup \{K_n\}_{n<-N}$ are pairwise different. $\square$

### Proof of Theorem 5.3

We will show that the reduced Khovanov homology of the knots $\{K_n\}_{|n|\ge N}$ are pairwise different by studying how the invariant ${\widetilde{{{\,\textrm{Kh}\,}}}}(T)$ pairs with the arc . First, suppose ${\widetilde{{{\,\textrm{Kh}\,}}}}(T)$ contains only rational components of slope 0: In this case Theorem 3.1 implies that *T* is horizontally split, contradicting the assumption in Theorem 5.3. Next, suppose ${\widetilde{{{\,\textrm{Kh}\,}}}}(T)$ only contains curves of slope 0 of which at least one is special: This case is covered by Lemma 5.4. The last case of ${\widetilde{{{\,\textrm{Kh}\,}}}}(T)$ containing curves of non-zero slopes is covered by Lemma 5.5. $\square$

### Non-trivial band detection

Joshua Wang asked if it is possible to recover his result [38, Theorem 1.1] using our techniques. The answer is yes:

#### Theorem 5.6

(Reformulation of Theorem 1.3) Suppose *T* is not horizontally split, and *T*(0) is a split link. Then the unoriented knots $\{K_n\}_{n\in {\mathbb {Z}}}$ are pairwise different.

The only missing ingredient in the proof is the following.

#### Theorem 5.7

(Split closure property) Suppose *T*(0) is a split link $K \cup K'$. Then ${\widetilde{{{\,\textrm{Kh}\,}}}}(T)$ only contains components of slope 0.

#### Proof of Theorem 5.6

Theorem 5.7 implies that ${\widetilde{{{\,\textrm{Kh}\,}}}}(T)$ only contains components of slope 0. By Theorem 3.1 we know that the tangle *T* must contain special components of slope 0. Lemma 5.4 now proves the statement. $\square$

#### Proof of Theorem 5.7

Placing the reduction point to the top-left end of *T*, we consider the reduced Khovanov homology of the split link ${\widetilde{{{\,\textrm{Kh}\,}}}}(T(0))={\widetilde{{{\,\textrm{Kh}\,}}}}(K\cup K')$. Since only one of the two components is reduced (say *K*), we may consider the basepoint action on ${\widetilde{{{\,\textrm{Kh}\,}}}}(K\cup K')$ with respect to a basepoint on $K'$, which we place near the bottom left end of *T*. Keeping in mind that we work with ${\mathbb {F}}$ coefficients, we have

$$\begin{aligned} {\widetilde{{{\,\textrm{Kh}\,}}}}(K\cup K')={\widetilde{{{\,\textrm{Kh}\,}}}}(K) \otimes _{{\mathbb {F}}} {{\,\textrm{Kh}\,}}(K') \end{aligned}$$

and the basepoint action of ${\mathbb {F}}[x]/(x^2)$ on ${\widetilde{{{\,\textrm{Kh}\,}}}}(K\cup K')$ is induced by the basepoint action of ${\mathbb {F}}[x]/(x^2)$ on ${{\,\textrm{Kh}\,}}(K')$. Over ${\mathbb {F}}$ the latter basepoint action on unreduced Khovanov homology is well-known to be free [30, Corollary 3.2.C], and so the basepoint action of ${\mathbb {F}}[x]/(x^2)$ on ${\widetilde{{{\,\textrm{Kh}\,}}}}(K\cup K')$ is also free.

**Fig. 13:**
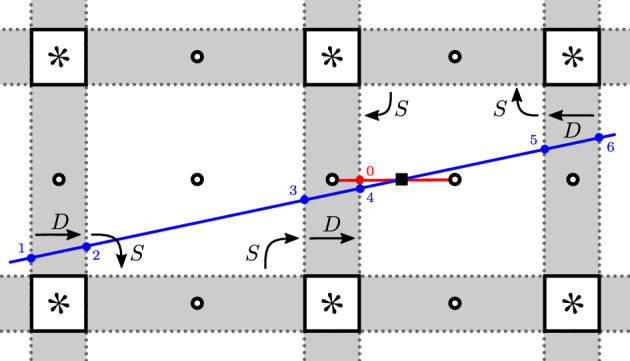
The lifts of the arc  and a rational or special curve $\gamma$ of sufficiently shallow positive slope, based at a common point of intersection

We now come back to the tangle *T* and leverage the description of Khovanov homology in terms of the Floer homology of curves:Let us suppose there is a curve  in  of slope $\nicefrac p q \ne 0$. By adding twists to the lower two punctures we may assume that $\nicefrac p q$ is positive and as close to 0 as we want. After pulling the curves tight in the planar cover, using Observation 2.11, each intersection between  and  locally looks as in Fig. 13.

Thus the complexes over the algebra ${\mathcal {B}}$ associated to the curves  and  are as follows:where the subscripts are used to simply label generators. According to [15, Theorem 1.5], the Lagrangian Floer homology between two curves is isomorphic to the homology of the morphism space between the corresponding complexes:As ${\mathbb {F}}[x]/(x^2)$-modules, they are direct summands ofsince  is a component of  and hence  is a direct summand of . Consider now a morphism consisting of a single arrow . (This morphism corresponds to the single intersection in Fig. 13.) The basepoint action multiplies all the labels of a morphism by ; see the discussion before [17, Lemma 6.46] for a detailed explanation of the basepoint action in the context of morphism spaces of chain complexes. Thus the basepoint action sends the morphism  to the morphism , which is null-homotopic; the null-homotopy is . Furthermore, the morphism  is not in the image of the basepoint action, because every morphism that is homotopic to the one in the image of the basepoint action cannot contain identity arrows $\xrightarrow {{{\,\textrm{id}\,}}}$. This is because both  and  correspond to the pulled tight curves, and thus do not contain any $\xrightarrow {{{\,\textrm{id}\,}}}$ in their differentials. We conclude that the morphism  represents torsion in the basepoint action of ${\mathbb {F}}[x]/(x^2)$ on ${\widetilde{{{\,\textrm{Kh}\,}}}}(T(0))$. So this action is not free, contradicting the fact that *T*(0) is split. $\square$

### Towards split closure detection

It is natural to wonder if the converse of Theorem 5.7 also holds.

#### Conjecture 5.8

(Split closure detection) Given a Conway tangle *T*, *T*(0) is a split link if and only if ${\widetilde{{{\,\textrm{Kh}\,}}}}(T)$ only contains components of slope 0.

In this direction, we can offer the following result:

#### Theorem 5.9

*T*(0) is a split link if ${\widetilde{{{\,\textrm{Kh}\,}}}}(T)$ only contains components of slope 0 and no rational curves of length greater than 1.

#### Proof

If ${\widetilde{{{\,\textrm{Kh}\,}}}}(T)$ only contains components of slope 0 then ${\widetilde{{{\,\textrm{Kh}\,}}}}(T(0))$ is isomorphic to the Lagrangian Floer homology between  and the rational components of ${\widetilde{{{\,\textrm{Kh}\,}}}}(T)$. If all rational components are (up to grading shift) equal to ${\textbf{r}}_1(0)$ this implies that the basepoint action on ${\widetilde{{{\,\textrm{Kh}\,}}}}(T(0))$ is free. So by the main result of [21], *T*(0) is a split link. $\square$

To prove Conjecture 5.8, it remains to show that if ${\widetilde{{{\,\textrm{Kh}\,}}}}(T;{\mathbb {F}})$ only contains curves of slope 0 then all its rational components have length 1. Note that it is important that we use coefficients in ${\mathbb {F}}$, because this statement is false away from characteristic 2. However, over ${\mathbb {F}}$, we in fact expect this to be a more general property of the multicurve invariants ${\widetilde{{{\,\textrm{Kh}\,}}}}$:

#### Conjecture 5.10

For any Conway tangle *T*, the length of any rational component of ${\widetilde{{{\,\textrm{Kh}\,}}}}(T;{\mathbb {F}})$ is equal to 1.
